# ASO Author Reflections: Reconsidering the Role of Ischemia and Surgical Approach in Functional Outcomes After Minimally Invasive Partial Nephrectomy

**DOI:** 10.1245/s10434-025-18681-z

**Published:** 2025-11-07

**Authors:** Pier Paolo Prontera, Antonio Balestra, Marco Lattarulo, Gianluigi Califano, Francesco Di Bello, Claudia Collà Ruvolo, Simone Morra, Angelo Porreca, Luca Di Gianfrancesco, Arman Tsaturyan, Francesco Dibenedetto, Francesco S. Grossi

**Affiliations:** 1 Department of Urology, “S.S. Annunziata” Hospital, Taranto, Italy; 2 Department of Urology, Hospital “Valle D’Itria”, Martina Franca, Italy; 3 Department of Neurosciences, Reproductive and Odontostomatologic Sciences, University of Naples “Federico II”, Naples, Italy; 4 Department of Urology, Humanitas Gavazzeni Hospital, Bergamo, Italy; 5 Department of Urology, Yerevan State Medical University after Mkhitar, Heratsi, Yerevan, Armenia; 6 Department of Urology Erebouni Medical Center, Yerevan, Armenia; 7 Master of Science in Nursing, Department of Operating Room, “S.S. Annunziata” Hospital, Taranto, Italy

## Past

Historically, the preservation of renal function after partial nephrectomy has been closely linked to ischemia management and surgical approach. Surgeons have long debated whether the clamp technique or warm ischemia time (WIT) directly determines postoperative renal recovery. The advent of robotic surgery appeared to further shift this paradigm, suggesting potential superiority in precision and nephron preservation. Yet, the specific contribution of tumor localization, ischemia type, and surgical modality remained unclear in patients with low-to-intermediate complexity cT1 renal tumors.^1,2^

## Present

Our multicenter study demonstrated that, when WIT is maintained below 30 min, neither ischemia type (on-clamp versus off-clamp) nor surgical approach (robotic versus laparoscopic) significantly affects renal functional outcomes up to 12 months postoperatively.^1^ Functional recovery, assessed through serum creatinine and estimated glomerular filtration rate (eGFR) trends, was comparable across all groups. These findings support the concept that residual vascularized parenchymal volume, rather than ischemia duration, plays a more decisive role in long-term renal preservation.^3^ This perspective emphasizes surgical precision and parenchymal conservation over the choice of ischemic technique. Similarly, the potential advantages of robotic assistance may be less evident in low-complexity tumors, where both robotic and laparoscopic approaches can achieve excellent outcomes in expert hands.^4,5^

## Future

Future prospective, larger-scale studies integrating volumetric imaging and quantitative parenchymal assessment are needed to define thresholds of ischemic tolerance and refine patient selection for clampless or robotic-assisted techniques. Functional outcomes should be evaluated within the broader framework of tumor complexity, surgeon experience, and individualized surgical planning, moving beyond a purely ischemia-centered model.^2,3^
